# Modelling the impact of sublingual immunotherapy versus subcutaneous immunotherapy on patient travel time and CO_2_ emissions in Sweden

**DOI:** 10.1038/s41598-024-51925-8

**Published:** 2024-01-18

**Authors:** Lars-Olaf Cardell, Thomas Sterner, Waqas Ahmed, Andreas Kallsoy Slættanes, Mikael Svärd, Richard F. Pollock

**Affiliations:** 1 Division of ENT Diseases, Department of Clinical Science, Intervention and Technology, Karolinska Institute, Stockholm, Sweden; 2 Department of Economics, School of Business, Economics and Law, University of Gothenburg, Gothenburg, Sweden; 3 Covalence Research Ltd, Harpenden, UK; 4 ALK, Bøge Allé 1, DK-2970 Hørsholm, Denmark; 5 ALK Nordic, Faktorvägen 9, SE-434 21 Kungsbacka, Sweden

**Keywords:** Climate change, Immunotherapy

## Abstract

In Sweden, allergy immunotherapy (AIT) is available as either subcutaneous immunotherapy (SCIT) injections or sublingual immunotherapy (SLIT) tablets and is used to treat moderate-severe allergic rhinitis (AR). This study sought to determine treatment-related CO_2_ emissions and travel times in Swedish patients receiving either SCIT or SLIT-tablets. A list of specialized Swedish AR clinics that administer AIT was determined, and respective co-ordinates retrieved. Swedish municipality population data were obtained from a national database. The mean distance from each Swedish municipality to the nearest AR clinic was calculated, adjusted using a detour index, and weighted by estimated patient population size. Transport modality data were obtained from a Swedish urban transport study and CO_2_ emissions were obtained from Government sources. The mean number of annual SLIT-tablets and SCIT doses required were calculated based on product labels and clinical expert input. The annual number of healthcare professional interactions were layered into the model to estimate changes in mean patient travel time, distance, and travel-related CO_2_ emissions associated with using SCIT versus SLIT-tablets. Mean annual travel-related CO_2_ emissions were 410 tonnes (to two significant figures [s.f.]; standard deviation [SD] 90) with SLIT-tablets, versus 1700 tonnes (SD 380) for SCIT, resulting in mean annual savings of approximately 1300 tonnes (SD 290) of CO_2_ if all AIT patients were to receive SLIT-tablets instead of SCIT, over 380 times greater than 2021 average Swedish CO_2_ emissions per capita. Approximate mean annual travel times for patients taking SLIT-tablets were 66,500 h (three s.f.; SD 14,400), and 278,000 h (SD 60,200) for SCIT, resulting in mean annual savings of 211,000 h (SD 45,800) if all AIT patients were to receive SLIT-tablets instead of SCIT. Compared with SCIT injections, SLIT-tablets led to substantial reductions in treatment-related CO_2_ emissions and travel times for Swedish patients.

## Introduction

Allergic rhinitis (AR) is an inflammatory disorder of the nasal mucosa that occurs due to allergen exposure^1^. Allergens may include pollens, molds, dust mites and animal dander^2^, with exposure triggering IgE-mediated inflammation^3^. The main symptoms of AR comprise nasal congestion, rhinorrhoea, postnasal drip, and nasal pruritis^4^. Symptoms of AR can subsequently result in sleep disturbance, fatigue, mood changes, and affected cognitive function, which can in turn significantly impact productivity and quality of life (QoL)^5^. Currently, the ARIA guidelines classify AR based on the duration and severity of symptoms and QoL^6^. Patients who experience one or more of the following symptoms: abnormal sleep, impairment of daily activities, sports and leisure, abnormal work and school, or troublesome symptoms, would fall into the ‘moderate-severe’ AR category^6^.

Worldwide, AR is estimated to impact 500 million people^6^, with the prevalence increasing over the second half of the twentieth century in many countries, including Sweden^7–9^, where the estimated prevalence of AR is 28%^10^. Given the significant proportion of the Swedish population suffering from AR, a 2016 Swedish study sought to investigate the annual AR-related costs per individual. The results demonstrated a significant economic burden, as mean annual direct and indirect AR-related costs per individual per year were €210.30 and €750.80, respectively, with presenteeism representing 70% of the total cost^11^. However, usage of conventional symptomatic therapies, including antihistamines, topical steroids, and nasal decongestants were high in the participant sample. This finding demonstrates that current symptomatic treatments may not be sufficient, particularly for patients with severe and persistent symptoms. Allergy immunotherapy (AIT) is an alternative, cost-effective therapy option that may be considered when symptomatic treatments are no longer sufficient^12,13^. This therapeutic approach involves building patients’ tolerance to allergens via repeated exposure to controlled doses of allergen extract, in order to improve symptoms associated with subsequent allergen exposure^14,15^.

Presently, AIT is the only disease-modifying treatment available for AR^16^ and can be delivered as subcutaneous immunotherapy (SCIT) injections and sublingual immunotherapy (SLIT) tablets or drops (with only tablets currently available in Sweden)^17^. Whilst both options demonstrate similar clinical efficacy in the long-term suppression of the allergic response^18^, the burden of their dosage schedules can differ significantly^19,20^. SCIT requires a course of injections that necessitate receiving treatment within a clinical setting, under the supervision of a healthcare professional (HCP)^21^. However, those receiving SLIT-tablets only require clinical supervision when taking their first dose (and when having annual clinical reviews), with subsequent doses being able to be taken from the patient’s home.

Due to the frequency of administration and clinical setting required for SCIT, patients often spend a significant amount of time travelling to receive treatment. From a healthcare payer’s perspective, these frequent patient interactions with the healthcare system can result in increased costs. Additionally, depending on the mode of transport used by patients receiving SCIT, frequent visits to and from clinics can have environmental effects. Specifically, these effects include increased CO_2_ (and CO_2_ equivalent) emissions related to treatment. In cases where patients live in rural areas and are required to travel long distances to their closest clinic, these emission effects can be substantial.

Given the significant impact that increased treatment-related CO_2_ emissions can have on wider society, there is a renewed interest in promoting sustainable health technologies that help to reduce patients’ carbon footprints. For example, the National Institute for Health and Care Excellence (NICE) have recently issued a patient decision aid on the use of asthma inhalers, which focuses on the difference in carbon footprint between metered dose and dry powder inhalers^22^.

The aim of this study was to determine the treatment-related CO_2_ emissions and travel times for patients receiving either SLIT-tablets or SCIT injections from their nearest AR clinic in Sweden. A bespoke algorithm used for this analysis was developed using Python software version 3.10.4, incorporating multi-modal transport matrices and, where possible, municipality-specific data inputs.

## Methods

### Swedish municipality co-ordinate data

A shapefile outlining the boundary co-ordinates for each of the 290 Swedish municipalities was first obtained from an online directory. The shapefile was imported for analysis using the GeoPandas 0.12 library in Python version 3.10.4.

### Clinic locations

A definitive list of specialized Swedish AR clinics that delivered both SCIT and SLIT-tablets was obtained from ALK-Abelló. The respective latitude and longitude co-ordinates (presented in decimal degrees format) for each of the clinics were then obtained via the Google Maps geocoding application programming interface (API). For each hospital-based clinic, one central location (usually the main entrance) was used to mimic routine travel practices for appointments, where patients would most likely arrive by their chosen mode of transport at the main hospital entrance, before finding their desired clinic on foot.

**Figure 1:**
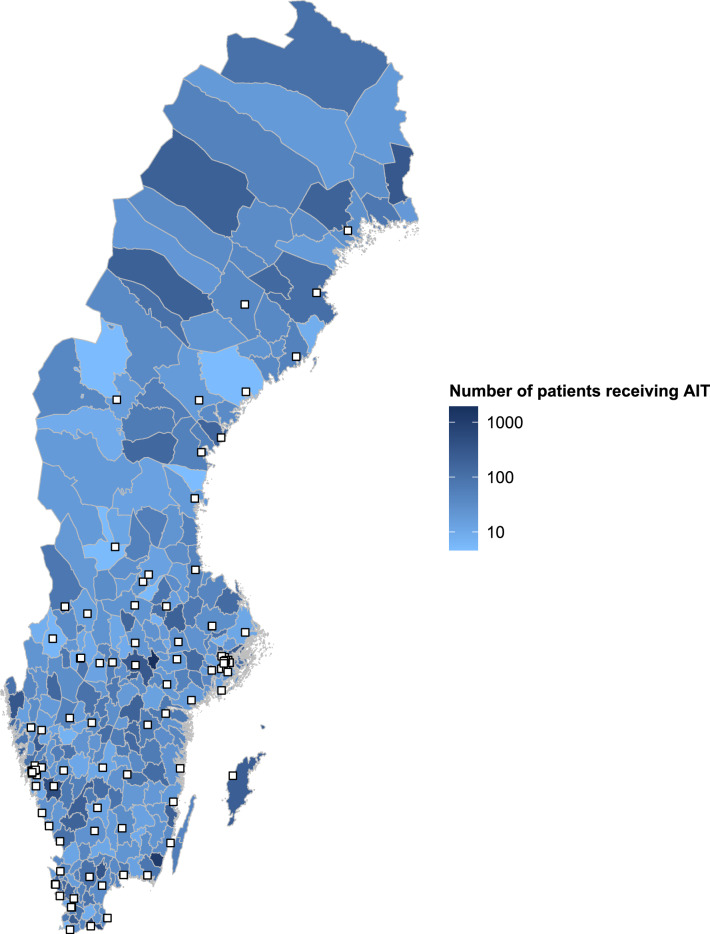
Map outlining the locations of AR clinics and density of AIT patient populations across Swedish municipalities. AIT, allergen immunotherapy. White squares represent individual AR treatment sites.

A total of 105 AR clinics were included in the final analysis. The co-ordinate data for each AR clinic and estimated AR patient population data for each municipality were then superimposed onto the shapefile map of Swedish municipalities using R version 4.2.1 (Fig. 1). A visual inspection of the image presented in Fig. 1 was conducted to ensure that the co-ordinate data had been correctly identified, recorded, and processed. Once the visual inspection was completed, the final AR clinic list, along with their respective co-ordinates, were imported into Python/GeoPandas data frames for analysis.

### Travel distance algorithm

An algorithm was developed in Python to first determine the representative point of each municipality, presented in terms of latitude and longitude. The representative point function determined the centrally calculated co-ordinate (central location) for each municipality based on pre-determined latitude and longitude data, whilst accounting for irregularities in municipality boundary polygons. Second, a nested ‘while loop’ was implemented to calculate the haversine distance (straight line distance over a spheroid) in kilometres from the central location of each municipality to each of the 105 AR clinics. A schematic of the algorithm is presented in the Supplementary Fig. S1.

### Detour index

As the haversine formula calculated the shortest ‘straight line’ distance from the municipality’s central location to the closest clinic over a spheroid (i.e., to account for the curvature of the Earth), a detour index was calculated and applied. The detour index is a measure of the deviation from the shortest possible route connecting two points required in order to travel between the points using a specific mode of transport^23^. Actual travel distances from each municipality’s central location to the nearest AR clinic were calculated, which were dependent on network routes of available transport modes (e.g., road networks for car travel).

The detour index was calculated as the distance travelled (e.g., driving distance based on road routes) divided by the straight-line distance (calculated using the haversine formula). A higher detour index therefore indicated larger deviations from the straight-line distance, whereas a lower detour index indicated reduced deviations from the straight-line distance. The distance travelled was determined using the Google Maps Distance Matrix API. Where multiple travel routes were available, the shortest route in terms of time travelled was selected to replicate the likely route selection process adopted by patients. Detour indices were only applied for transport modes using road networks (e.g., bus, car, and motorcycle); limited network data were available for ferry and train routes, and a detour index was not applied to these modes of transport.

Given the heterogenous terrains within Sweden, two separate average detour indices and standard deviations (SDs) were calculated. One mean index was calculated for 15 ‘mountainous’ municipalities, and a second mean detour index was calculated for the remaining 275 ‘non-mountainous’ municipalities^24^. In both cases, a selection of randomly generated travel routes from the most populous municipality were chosen to calculate mean detour indices and SDs. The final mean detour index for mountainous municipalities was 1.90 (SD 0.46), versus 1.71 (SD 0.50) for non-mountainous municipalities. An illustration of the straight-line (haversine) versus travelled distances for randomly-generated travel routes in Stockholm and Gällivare are presented in Supplementary Figs. S2 and S3, respectively.

The detour indices were sampled in the main travel distance algorithm. Specifically, the travel distance algorithm was run for 1000 iterations, with a new detour index for mountainous and non-mountainous municipalities sampled from a normal distribution prior to each iteration. Samples leading to a detour index value of less than 1 were resampled (repeatedly if necessary) to eliminate the possibility of modelling routes shorter than the straight-line distance. The shortest distances from each municipality’s representative point to the nearest clinic were then calculated and multiplied by the relevant detour index, to determine a more accurate estimate for the distance travelled. Finally, estimates of CO_2_ emissions and travel times from each of the 1000 iterations were compiled, with the mean and SD of these overall results then calculated and reported.

### Swedish population data

Population data for each Swedish municipality were obtained from the Official Statistics of Sweden database^25^, with data updated to include figures for March 2022. The data were cleaned before being imported into Python for analysis.

### Transport modality matrix

Mode of transport usage data in Sweden were obtained from Kenworthy et al.^26^, which presented a detailed outline of the urban transport eco-urbanism characteristics of the Stockholm, Malmö, Göteborg, Linköping, and Helsingborg urban regions in southern Sweden, in addition to national level data. For the present analysis, “kilometre per capita” data and average speeds for relevant modes of transport were extracted, before being used to calculate the individual proportions of patients who would use each mode of transport across Sweden. These proportions were then multiplied by the relevant patient cohort figures, to yield a final number of patients with moderate-severe AR who would use each mode of transport. Municipality-specific data for Stockholm, Helsingborg, Linköping, Malmö and Göteborg, were also incorporated into the final analysis to allow an increased level of specificity for these more populous, urban areas of Sweden.

The final modes of transport that were used within the Python algorithm were bus, car, ferry, motorcycle, and train. A summary of the transport matrices used for the final analysis is presented in Table 1.

**Table 1:** Transport modality matrices.

Transport mode	Percentage of patients using specified transport modes (%)					
	Sweden	Malmö	Stockholm	Göteborg	Linköping	Helsingborg
Bus	9.60	6.24	8.87	13.88	6.96	11.39
Car	78.44	81.90	71.56	72.45	87.82	80.56
Ferry	0.08	0.00	0.19	0.21	0.00	0.00
Motorcycle	0.74	0.72	0.62	0.87	0.74	0.77
Train	11.14	11.14	18.76	12.60	4.47	7.28

### One-year cohort patient group

For the main analyses, a 1-year ‘snapshot’ cohort was determined, whereby the total number of patients with moderate-severe AR receiving AIT in any 1 year was identified to be 20,330^27^. This figure comprised 11,730 patients receiving SLIT-tablets and 8600 patients receiving SCIT. All patients in the cohort were assumed to receive either SCIT or SLIT-tablets, to determine the maximum potential impact of each treatment type on treatment-related CO_2_ emissions and travel times. For SCIT, the cohort was further divided into a titration group (n = 1952) and a maintenance group (n = 18,378). The titration group comprised patients who were newly diagnosed with moderate-severe AR, and was calculated using annual incidence data^28^. These patients would receive weekly, increasing doses of SCIT over a defined timeframe (e.g., 7 or 15 weeks). The maintenance group comprised patients who had completed a previous titration regime, and were now receiving less frequent, fixed-doses of SCIT.

### Three-year cohort patient group

An additional analysis was conducted which considered a hypothetical 3-year ‘incident cohort’. Here, patients who had recently developed moderate-severe AR at the beginning of year one and were being prescribed AIT were followed over a 3-year treatment period. A 3-year treatment period was chosen as this timeframe was outlined in the relevant literature as being required for AIT to achieve the desired long-term modifying effect^29–32^. Given the hypothetical nature of this cohort, a patient sample of 1000 was assumed. This figure was not intended to be reflective of the patient numbers found in routine clinical practice, but rather to aid in demonstrating the travel impacts of AIT for a comprehensible patient sample size. Once again, for this cohort all patients were assumed to be taking either SCIT or SLIT-tablets. Results for this cohort were reported on a per-patient basis, to demonstrate the likely treatment-related CO_2_ emissions and travel times for one patient undergoing one ‘full’ cycle of either SCIT or SLIT-tablets.

### Supplementary patient funnel

Current recorded figures for patients with moderate-severe AR receiving AIT may not account for potential factors that may lead to an under-estimation of actual patient numbers. Barriers to receiving treatment can be one factor, with some patients being limited to medication access due to travel logistics or financial constraints. Patients may also be unaware of the medical options available to them, and subsequently not seek further treatment other than conventional allergy therapies, despite possibly benefitting from AIT. Given this, a patient funnel was developed to illustrate the overall likely prevalence of moderate-severe AR in Sweden, and therefore, the potential national need for AIT. While the patient funnel was not used within the main analyses, the figures were determined to estimate how widely AIT could potentially be used across the Swedish moderate-severe AR population, if all eligible patients had access to treatment. Details of the supplementary patient funnel figures can be found in the “Discussion” section.

### Treatment schedule: SCIT

Treatment schedules for both titration and maintenance regimes of SCIT were sourced from a relevant Summary of Product Characteristics (SmPC) document^20^. An independent clinical expert was then consulted to determine the likely use of the selected treatment schedules in routine practice, specifically with regard to the different titration regimes. The results from this consultation showed that 7- and 15-week titration regimes were most likely to be used in clinical practice. Therefore, these regimes were used in our analysis for patients who were assigned to the titration-regime group (a full breakdown of these schedules is presented in Supplementary Table S1).

### Treatment schedule: SLIT-tablets

Similarly, dosage schedules for SLIT-tablets were taken from a relevant SmPC document^19^. Based on this data, input from an independent clinical expert, and AIT guidelines, it was determined that patients in the 1-year snapshot cohort receiving SLIT-tablets would be required to take one supervised dose and attend one physical follow-up appointment annually^33^. This was assuming that all patients receiving SLIT-tablets were in their first year of therapy. For the 3-year incident cohort, it was established that patients receiving SLIT-tablets would be required to take one supervised dose within the first year and attend three additional follow-up appointments over the 3-year period.

### CO_2_ emissions data

CO_2_ emissions data were obtained from the United Kingdom (UK) Department for Business, Energy & Industrial Strategy^34^. This data source described the CO_2_ equivalent emissions of the relevant transport modes in grams per passenger kilometre. Although Swedish-specific data were not available, UK-specific data were used as a proxy as these allowed for consistent reporting for all relevant transport modes included within our analyses. The final CO_2_ emission data used for each transport type can be found in Supplementary Table S2.

## Results

### Travel distances

**Figure 2:**
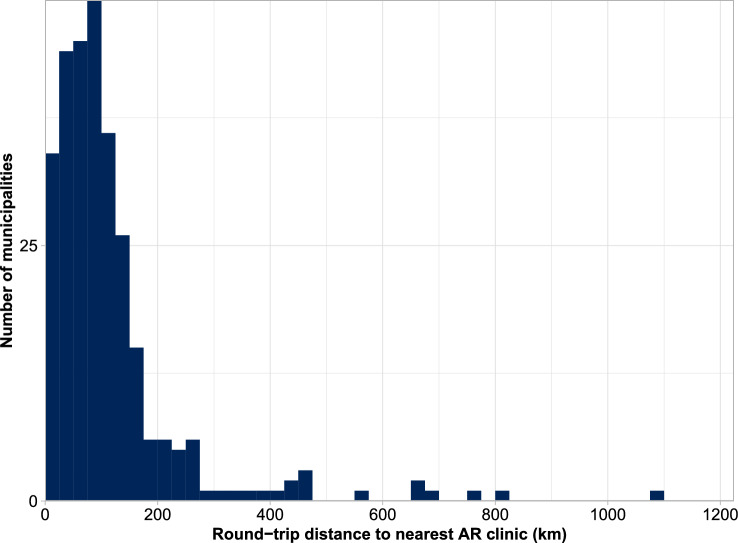
Summary of round-trip distances across all 290 municipalities. AR, allergic rhinitis; km, kilometre.

Over 1000 iterations, the unweighted mean round-trip distance across the 290 municipalities was 117.5 km. The AIT-treated population-weighted mean round-trip distance was 58.2 km, substantially lower than the unweighted mean as the majority of patients resided in urban municipalities, where AR clinics were more abundant and therefore likely closer to places of residence than in rural municipalities (Fig. 1). The longest round-trip distance was 1089.9 km, from the centre of Kiruna to Sunderbyn hospital. The shortest round-trip distance was 3.8 km, from the centre of Nacka to Nacka Hospital. The majority of municipalities had a round-trip distance to the nearest clinic below 200 km. A summary of the travel distances across the 290 municipalities can be found in Fig. 2, with detailed data for each municipality presented in Supplementary Table S3.

### One-year cohort

**Figure 3:**
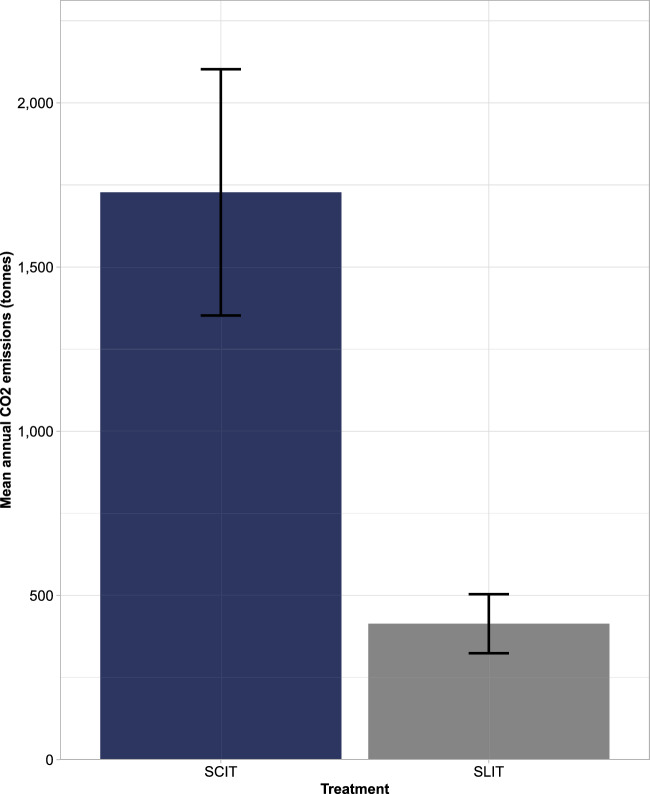
Mean annual CO_2_ emissions: 1-year cohort. SCIT, subcutaneous allergy immunotherapy; SLIT, sublingual allergy immunotherapy.

**Figure 4:**
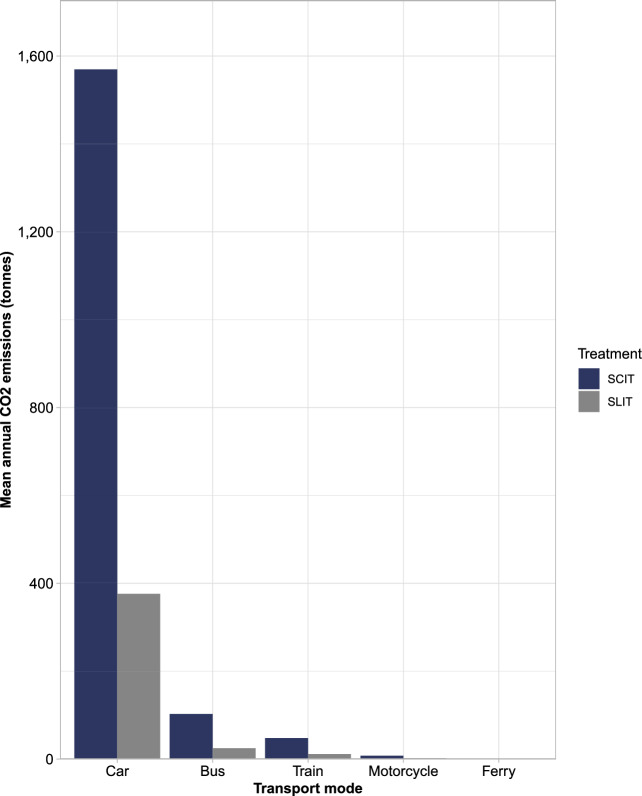
Mean annual CO_2_ emissions by transport mode: 1-year cohort. SCIT, subcutaneous allergy immunotherapy; SLIT, sublingual allergy immunotherapy.

Over 1000 iterations, the mean total annual treatment-related CO_2_ emissions if all patients were taking SLIT-tablets was 414 tonnes (SD 90), versus 1728 tonnes (SD 375) for SCIT (a ratio of 1:4.2). This resulted in mean potential annual savings of 1314 tonnes (SD 285) of CO_2_ if all patients were to receive SLIT-tablets instead of SCIT (Fig. 3). This saving represented 384 times the average Swedish CO_2_ emissions per capita for 2021^35^. In terms of transport modality, most annual treatment-related CO_2_ emissions stemmed from car usage (376 tonnes [SD 82] for SLIT-tablets and 1570 tonnes [SD 341] for SCIT). Buses and trains were the second and third biggest contributors to treatment-related CO_2_ emissions, respectively, with ferry usage being the lowest contributor (Fig. 4).

**Figure 5:**
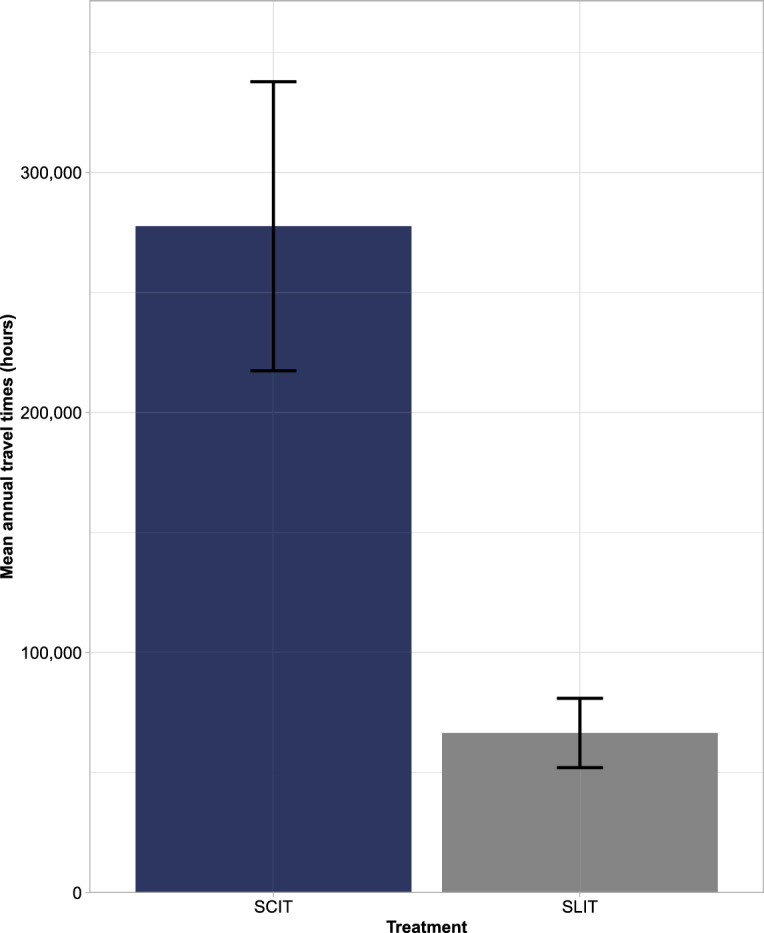
Mean annual travel times: 1-year cohort. SCIT, subcutaneous allergy immunotherapy; SLIT, sublingual allergy immunotherapy.

**Figure 6:**
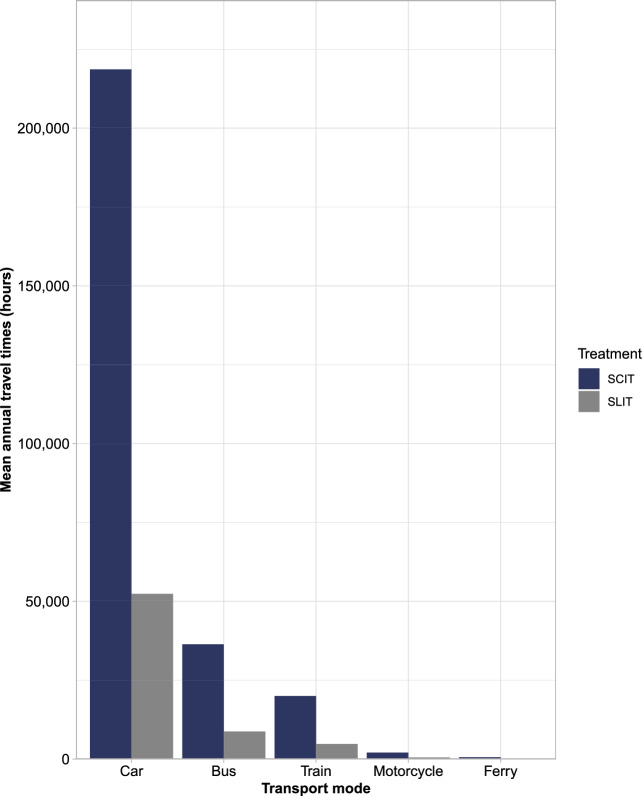
Mean annual travel time by transport mode: 1-year cohort. SCIT, subcutaneous allergy immunotherapy; SLIT, sublingual allergy immunotherapy.

The mean total annual treatment-related travel time if all patients were taking SLIT-tablets was equal to 66,544 h (SD 14,433), and 277,621 h (SD 60,216) for SCIT (again a ratio of 1:4.2). This resulted in mean potential annual savings of 211,077 h (SD 45,783) if all patients were to receive SLIT-tablets instead of SCIT (Fig. 5). In terms of modality, the majority of treatment-related travel times stemmed from car usage (52,396 h [SD 11,367] for SLIT-tablets and 218,594 h [SD 47,422] for SCIT). Again, bus and train journeys were the second and third biggest contributors to treatment-related travel times, respectively, with ferry usage being the lowest contributor (Fig. 6).

### Three-year cohort

Mean total treatment-related CO_2_ emissions per patient taking SLIT-tablets were equal to 41 kg (SD 9) over the 3-year treatment period, and 311 kg (SD 67) for SCIT (a ratio of 1:7.6). This resulted in mean potential CO_2_ emission savings of 270 kg (SD 59) over one complete treatment period, for each patient who was to receive SLIT-tablets instead of SCIT.

Mean treatment-related travel times per patient taking SLIT-tablets were equal to 7 h (SD 1) over the 3-year treatment period, and 50 h (SD 11) for SCIT (a ratio of 1:7.1). This resulted in a mean potential travel time saving of 43 h (SD 9) over one complete treatment period, for each patient who was to receive SLIT-tablets instead of SCIT.

## Discussion

This study sought to determine the likely savings associated with treatment-related CO_2_ emissions and patient travel times when considering SLIT-tablets as an alternative to SCIT for patients with moderate-severe AR in Sweden. To our knowledge, this is the first study that attempts to evaluate the treatment-related CO_2_ emissions and travel times associated with different forms of AIT in any geographical setting. However, existing studies have investigated the carbon and environmental footprint of global healthcare generally^36^, with some studies placing specific focus on national services, such as the NHS^37^.

The analysis determined that SLIT-tablets would result in fewer visits to AR clinics, and subsequently a reduction in treatment-related CO_2_ emissions and travel times for patients experiencing moderate-severe AR. Specifically, for a given 1-year snapshot of 20,330 moderate-severe AR patients, SCIT was associated with approximately 1700 tonnes (to two significant figures [s.f.]; SD 380) of treatment-related CO_2_ emissions, and 278,000 h (three s.f.; SD 60,200) of patient travel time. SLIT-tablets, however, were associated with approximately 410 tonnes (two s.f.; SD 90) of treatment-related CO_2_ emissions and 66,500 h (three s.f.; SD 14,400) of patient travel time. If all patients receiving SCIT were to be instead given SLIT-tablets, this would represent annual savings of approximately 1,300 tonnes (two s.f.; SD 290) of CO_2_ emissions, and 211,000 h (three s.f.; SD 45,800) of patient travel time. Similarly, when considering the savings over a 3-year treatment course, for each patient who was to receive SLIT-tablets instead of SCIT, there would be potential savings of approximately 270 kg (SD 59) of CO_2_ emissions and 43 h (SD 9) of patient travel time.

The presente study had a number of strengths; the use of data on individual Swedish municipalities allowed for a high level of detail within the analysis, specifically when considering geographical differences associated with travel distances. Municipalities based in south or south-eastern, urban regions of Sweden had a higher density of AR clinics, resulting in shorter overall travel distances for treatment. However, those municipalities located in the north and north-western, more rural regions had a much lower density and overall availability of nearby AR clinics. In turn, although fewer patients with moderate-severe AR lived within the rural municipalities, they contributed disproportionately to both overall treatment-related CO_2_ emissions and travel times.

Another strength of the study was the use of transport modality data specific to Sweden, including the use of municipality-specific data for Stockholm, Helsingborg, Linköping, Malmö and Göteborg. The use of these region-specific inputs is likely to increase the accuracy of the final results and could also facilitate future comparisons of travel patterns between the five municipalities. The method used to determine average detour indexes for mountainous and non-mountainous municipalities also resulted in an analysis more closely tailored to the Swedish setting, increasing the likelihood that the results are representative of the Swedish AR population treated with AIT. The sampling approach used in the main travel distance algorithm also allowed the degree of uncertainty in the results to be characterized.

Similarly, based on the SmPC of SCIT and SLIT-tablets and input from independent clinical experts practicing in the field of AR, final patient funnels and dosage regimes for both SCIT and SLIT-tablets were developed. The incorporation of multiple evidence sources including local expert opinion into the modelling is likely to have increased the accuracy and relevance of outcomes for patients receiving AIT in routine practice in Sweden. Furthermore, the use of both 1- and 3-year patient cohorts, in addition to the reporting of patient-level data on CO_2_ emissions and travel times in the latter cohort, allow the results to be interpreted from multiple perspectives and utilised by multiple stakeholders.

One limitation of the study was the lack of transport-mode-specific CO_2_ emissions data from Sweden. Given this, a European proxy dataset was sourced from the UK government. Although this data source may not be representative of Swedish transport infrastructure, it allowed for consistent reporting of emissions data across the included modes of transport and was calculated using robust and rigorous methods. Relatedly, CO_2_ emissions arising from the production of SCIT and SLIT-tablets were not incorporated, as the analysis focused exclusively on CO_2_ emissions arising from patient transportation. Furthermore, patient travel time and CO_2_ emissions resulting from routine pharmacy visits to collect SLIT-tablet prescriptions were not captured; however, there are significantly more retail pharmacy outlets in Sweden than specialized AR clinics^38^. The majority of patients would therefore be able to retrieve their SLIT-tablet prescriptions from pharmacies located near their place of residence or work, greatly increasing the possibility for opportunistic collection (i.e., attending the pharmacy alongside other errands), while also increasing the viability of using non-CO_2_ emitting modes of transport such as cycling or walking. Consequently, these omitted travel times and CO_2_ emissions would be unlikely to affect the final results significantly, although future studies could build on these findings by also incorporating routine pharmacy visits.

Another limitation was the assumption made regarding adherence to treatment. In the analysis, all patients were assumed to have a 100% adherence rate to their treatment, regardless of whether this comprised SCIT or SLIT-tablets. This assumption allowed the maximum likely treatment-related CO_2_ emissions and patient travel times for SCIT and SLIT-tablets to be modelled; however, these results may not be representative of outcomes in routine practice. Factors such as seasonal differences in transport modality usage, non-CO_2_ emitting modes of transport, and seasonal variations in AIT usage were not explored due to a lack of data identified in the literature.

**Figure 7:**
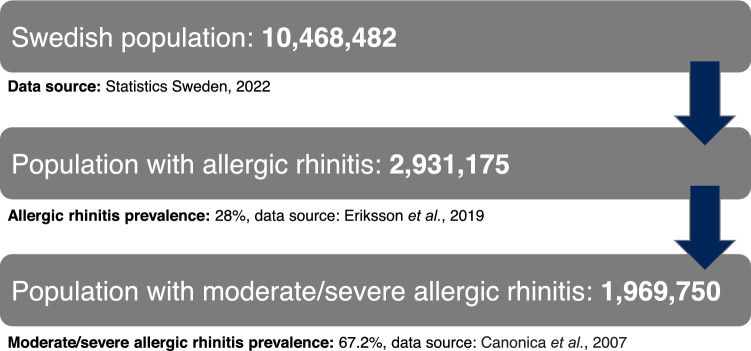
Supplementary patient funnel.

It should also be noted that, in routine clinical practice, general practitioners (GPs) can prescribe SLIT-tablets in Sweden. As a result, not all patients with moderate-severe AR may be required to travel to specialized AR clinics for their therapy initiation or annual follow up appointments. Consequently, travel time and CO_2_ emissions estimates for patients receiving SLIT-tablets may represent an over-estimation. Furthermore, while 20,330 patients were determined to be receiving AIT on an annual basis, this number is likely to be a substantial under-estimation of the actual eligible patient population both at present and in the future. Evidence that strengthens these assertions include the results of the calculated supplementary patient funnel, as well as rising trends in AR prevalence data from the literature^28,39–41^. Regarding the supplementary patient funnel, the total number of potential moderate-severe AR patients across Sweden was calculated to be 1,969,750; the full patient funnel and data sources^10,25,42^ are presented in Fig. 7. This number demonstrates that a substantially larger patient cohort may be eligible for AIT than the cohort used in our analysis. Therefore, potential CO_2_ emissions and travel time savings of SLIT-tablets versus SCIT could in-turn be significantly higher if all moderate-severe AR patients in Sweden had access to AIT.

Another factor contributing to the likely under-estimation is the additional travel limitations and logistical difficulties that patients may face when seeking treatment; for instance, where patients in more rural municipalities such as Kiruna would be required to travel over 1000 km for one AIT appointment. In reality, these patients would be unlikely to seek treatment requiring multiple clinic visits, which contrastingly highlights how calculated travel times and CO_2_ emissions for rural municipalities may be unlikely to occur in routine clinical practice. Patients living closer to AR clinics could also be more likely to opt for AIT (and indeed SCIT) than those patients living further away from the clinics, who would feel a more significant impact from travelling for treatment. However, these considerations would be unlikely to significantly impact the overall results presented here, as only a negligible proportion of the moderate-severe AR population in Sweden reside in rural municipalities (Supplementary Table S3).

A further limitation regarding our analysis approach was the assumption that all 20,330 patients receiving AIT in the 1-year cohort would receive either SCIT or SLIT-tablets. In reality, not all patients receiving SCIT can be transferred to SLIT-tablets due to the following reasons: AIT formulations for some allergies are only available as SCIT, contraindications, and adverse events profiles for each administration route. However, only a small proportion of patients receiving SCIT would be unable to receive SLIT-tablets, and this limitation is therefore unlikely to have a significant impact on the final results.

Finally, although the results of this study are specific to the Swedish setting, they are likely to be applicable across a variety of countries, given that AIT is widely used in Western Europe and the United States^43^. An increased global awareness of the impact of CO_2_ emissions could mean that a greater focus is placed on determining the environmental impacts, and indeed benefits, of forthcoming health technologies. Therapies such as SLIT-tablets, which involve minimal patient interaction with the healthcare system, could save both patients and the healthcare system significant financial outlay. Furthermore, a reduction in treatment-related CO_2_ emissions and travel times could have wider societal health benefits, particularly when considering the impacts of air pollution on patients with respiratory disease^44–46^.

This study estimated the maximum possible CO_2_ emissions and patient travel times for SCIT and SLIT-tablets. Results showed that significant savings could be made if patients were to receive SLIT-tablets instead of SCIT. While the per-patient estimates presented here are likely to be accurate for Sweden, future studies could further investigate the proportion of patients currently receiving either SCIT or SLIT-tablets in routine practice to better reflect the overall, population-level differences versus current practice.

## Conclusion

Compared with SCIT injections, SLIT-tablets reduced the number of visits required to AR clinics over a defined treatment period. This in turn led to significant reductions in treatment-related CO_2_ emissions and patient travel times.

### Supplementary Information

Supplementary Information.

## Data Availability

The raw data supporting the conclusions of this article will be made available by the authors, without undue reservation.
