# Supplementary material for: Modelling the impact of sublingual immunotherapy versus subcutaneous immunotherapy on patient travel time and CO2 emissions in Sweden
Source: Sci Rep. 2024 Jan 18;14:1575. doi: 10.1038/s41598-024-51925-8 (PMC10796394; doi:10.1038/s41598-024-51925-8)
Supplement: Supplementary file 1 — Supplementary Information. [file 41598_2024_51925_MOESM1_ESM.pdf]

Modelling the Impact of Sublingual Immunotherapy versus Subcutaneous Immunotherapy on Patient Travel Time and CO_2 Emissions in Sweden

Lars-Olaf Cardell[1†] (Orchid: 0000-0003-0538-9580), Thomas Sterner[2†] (ORCiD: 0000-0003-4771-3545), Waqas Ahmed[3](ORCiD: 0000-0002-9035-7614), Andreas Kallsoy Slættanes[4], Mikael Svärd[5], Richard F. Pollock[3,] * (ORCiD: 0000-0002-9873-7507)

[1] Division of ENT Diseases, Department of Clinical Science, Intervention and Technology, Karolinska Institute, Stockholm, Sweden

[2] Department of Economics, School of Business, Economics and Law, University of Gothenburg, Gothenburg, Sweden

[3] Covalence Research Ltd, Harpenden, UK

[4] ALK, Bøge Allé 1, DK-2970, Hørsholm, Denmark

[5] ALK Nordic, Faktorvägen 9, SE-434 21, Kungsbacka, Sweden

† These authors contributed equally to this work and share first authorship

*Correspondence

Name: Richard F. Pollock

E-mail: pollock@covalence-research.com

Supplementary material

Algorithm Description

To conduct this analysis, a variable (shortestDistance) was initialised to the maximum integer size in Python (sys.maxint). The algorithm was then implemented as a nested loop; in the first iteration of both loops (to calculate the distance from the first specialized AR clinic [c0] to the central location of the first municipality [m0]), if the distance in km calculated (x_{c0m0}) was smaller than the shortestDistance value (sys.maxint), the new value of shortestDistance was set to x_{c0m0}. For the second inner loop iteration (distance from the second clinic [c1] to the central location of the first municipality[m0]), if the distance in km calculated (x_{c1m0}) was smaller than shortestDistance, shortestDistance was updated to x_{c1m0} (by implication, where the newly-calculated distance in km (x_{c1m0}) was larger than x, the value of shortestDistance was retained).

This process was repeated, with the distance in km calculated from each of the 105 AR clinics (c0-c104) to the central location of the first municipality (m0), until the shortest possible distance calculated from the 105 AR clinics to the municipality central location was stored in the shortestDistance.

Once the process for the first municipality was complete, the final shortestDistance value was recorded in a new data frame, alongside the corresponding clinic name that resulted in the final shortestDistance and the name of the first municipality. The nested while loop then repeated the above processes for each of the remaining municipalities (m1-m289), updating the data frame with each shortestDistance value and associated data. A flowchart of the algorithm is presented in **Supplementary Figure S1**. Each of the Swedish municipalities, alongside data for their nearest AR clinic, population of AIT patients, and round trip distances to said AR clinic are presented in **Supplementary Table S3**.

Supplementary Figures

Supplementary Figure S1. Python algorithm flowchart

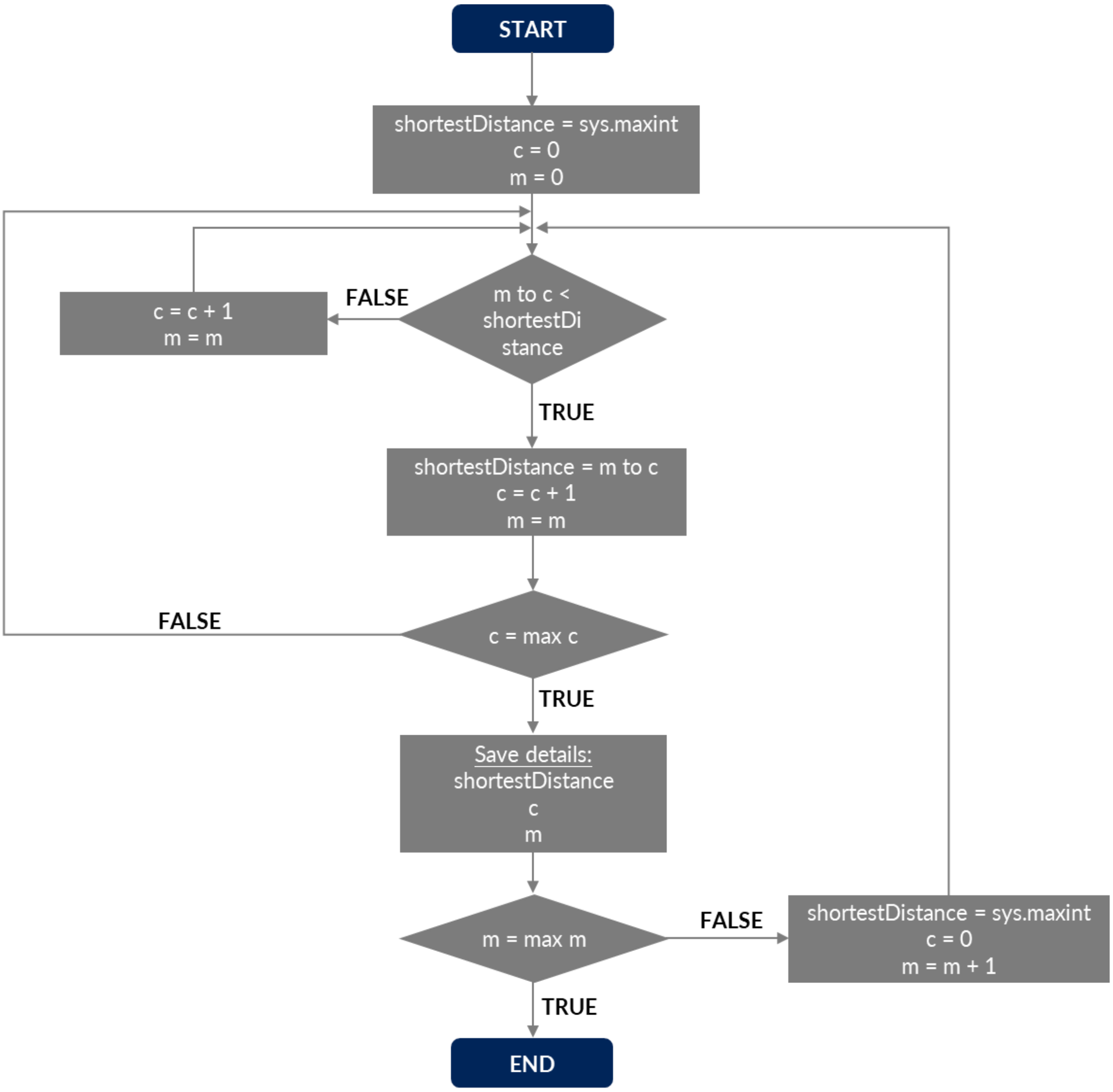

Variables: c, a specific AR clinic; m, central location for a specific municipality; shortestDistance, records the distance from the nearest clinic to each municipality's central location, max c, final clinic within loop analysis; max m, final municipality within loop analysis; sys.maxint, largest possible integer in Python.

Note: The flowchart assumes that the distance from the municipality's central location (m) to at least one clinic (c) is lower than the original shortestDistance value (sys.maxint).

Supplementary Figure S2. Haversine distances (grey) and actual routes (coloured) used in the calculation of a detour index for Stockholm municipality

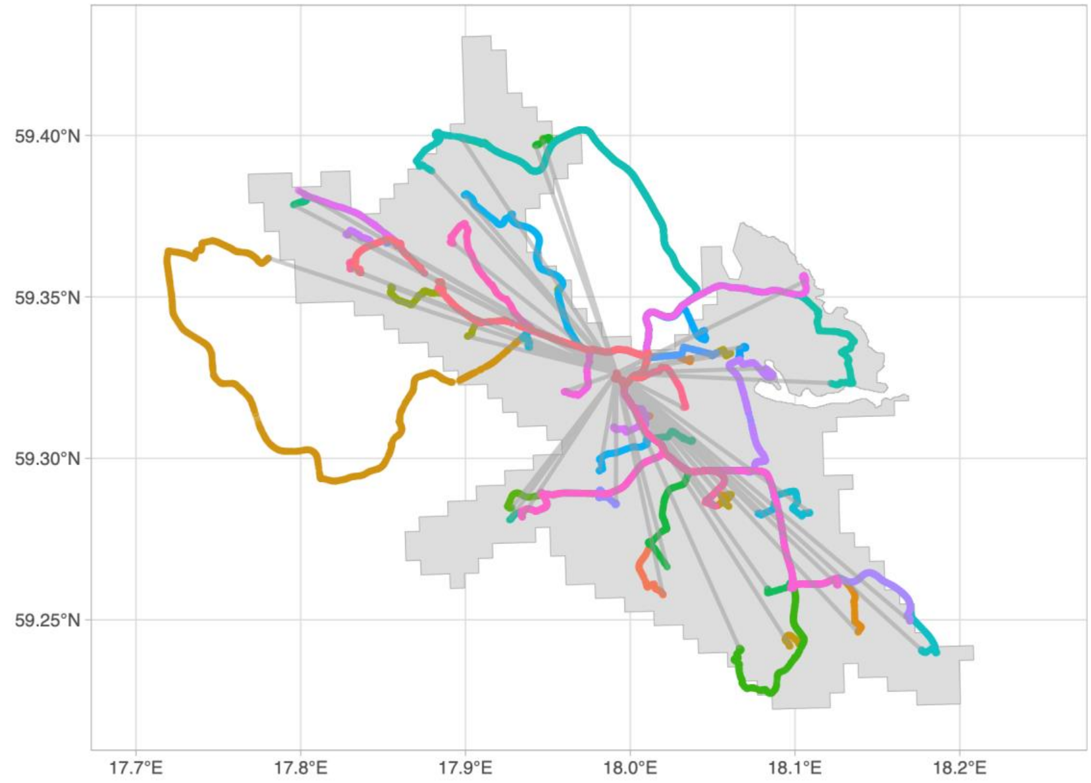

Supplementary Figure S3. Haversine distances (grey) and actual routes (coloured) used in the calculation of a detour index for Gällivare municipality

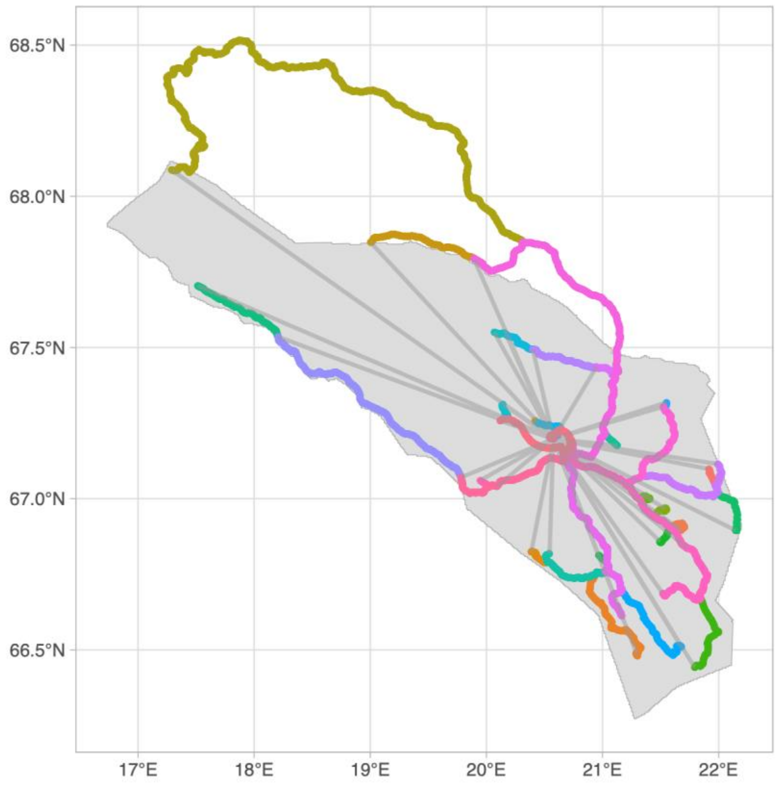

Supplementary Tables

Supplementary Table S1. Schedules for selected SCIT regimes

SCIT injection schedule	Titration schedule	Maintenance schedule
7-week titration (one-year cohort)<br>14 doses	One dose per week:<br>Weeks 1-7	Transition injection 1:<br>Week 9<br>Transition injection 2:<br>Week 13<br>Transition injection 3:<br>Week 19<br>Maintenance injections (taken every 8 weeks):<br>Weeks 27-52
15-week titration (one-year cohort)<br>21 doses	One dose per week:<br>Weeks 1-15	Transition injection 1:<br>Week 17<br>Transition injection 2:<br>Week 21<br>Transition injection 3:<br>Week 27<br>Maintenance injections (taken every 8 weeks):<br>Weeks 35-52
7-week titration (three-year cohort)<br>27 doses	One dose per week:<br>Weeks 1-7	Transition injection 1:<br>Week 9<br>Transition injection 2:<br>Week 13<br>Transition injection 3:<br>Week 19<br>Maintenance injections (taken every 8 weeks):<br>Weeks 27-156

SCIT injection schedule	Titration schedule	Maintenance schedule
15-week titration (three-year cohort)<br>34 doses	One dose per week:<br>Weeks 1-15	Transition injection 1:<br>Week 17<br>Transition injection 2:<br>Week 21<br>Transition injection 3:<br>Week 27<br>Maintenance injections (taken every 8 weeks):<br>Weeks 35-156

Abbreviations: SCIT, Subcutaneous allergy immunotherapy.

Supplementary Table S2. CO_2 emissions per transport mode

Transport mode	CO_2 emissions (g/km)
Bus	104
Car	192
Ferry	18
Motorcycle	100
Train	41

Supplementary Table S3. Swedish municipalities, their nearest AR clinic, population of AIT patients, and round-trip distances to said AR clinic

**Municipality**	**Nearest specialized AR clinic**	**Population receiving AIT for AR***	**Round-trip distance to clinic (km)****
Kiruna	Sunderbyn	44	1089.9
Arjeplog	Lycksele	5	804.7
Jokkmokk	Sunderbyn	9	759.5
Gällivare	Sunderbyn	34	683.3
Storuman	Lycksele	11	656.2
Sorsele	Lycksele	5	568.2
Pajala	Sunderbyn	12	672.7
Dorotea	Lycksele	5	474.4
Strömsund	Östersunds sjukhus	22	473.0
Vilhelmina	Lycksele	13	472.1
Härjedalen	Östersunds sjukhus	20	442.6
Älvdalen	Mora	14	431.2
Åre	Östersunds sjukhus	24	361.5
Arvidsjaur	Lycksele	12	415.9
Övertorneå	Sunderbyn	8	393.9
Krokom	Östersunds sjukhus	30	272.7
Överkalix	Sunderbyn	6	325.9
Malung	Mora	20	238.4
Haparanda	Sunderbyn	18	303.9
Berg	Östersunds sjukhus	14	221.7
Ånge	Sundsvalls sjukhus	18	293.3
Strömstad	Uddevalla sjukhus	26	265.4
Ljusdal	Hudiksvalls sjukhus	36	261.8
Bengtsfors	Uddevalla sjukhus	18	253.3
Malå	Lycksele	6	252.3
Ovanåker	Mora	23	251.2
Dals-Ed	Uddevalla sjukhus	9	240.6
Åsele	Lycksele	5	228.4
Bjurholm	Norrlands US	5	227.5
Piteå	Sunderbyn	82	225.9
Ragunda	Sollefteå sjukhus	10	219.1
Bräcke	Östersunds sjukhus	12	214.2
Älvsbyn	Sunderbyn	15	204.4
Åmål	Lidköpings Sjukhus	24	203.4
Bollnäs	Hudiksvalls sjukhus	52	201.6
Kalix	Sunderbyn	31	199.0
Vansbro	Hagfors VC	13	191.1
Norsjö	Lycksele	8	185.1
Boden	Sunderbyn	55	182.3

Municipality	Nearest specialized AR clinic	Population receiving AIT for AR*	Round-trip distance to clinic (km)**
Ödeshög	KSS Allergimott (ÖNH, Barn)	10	179.7
Askersund	Regionsjuk. Örebro ÖNH	22	178.9
Söderhamn	Hudiksvalls sjukhus	49	173.6
Vimmerby	Höglandssjukhuset	30	170.3
Östhammar	Enköpings barnspec mott	43	166.5
Emmaboda	Länssjukhuset i Kalmar	18	165.3
Valdemarsvik	Västerviks lasarett	15	164.9
Vadstena	US i Linköping/Allergi Centrum	15	163.6
Nordmaling	Norrlands US	14	161.9
Laxå	Karlskoga Lasarett	11	160.6
Vindeln	Lycksele	11	160.4
Rättvik	Mora	22	155.4
Tanum	Uddevalla sjukhus	25	155.4
Kinda	US i Linköping/Allergi Centrum	20	155.3
Tierp	Länssjuk. Gävle/Sandviken	42	155.0
Torsby	Torsby Sjukhus	22	154.0
Uppvidinge	Centrallasarettet i Växjö	18	153.5
Gullspång	Kristinehamns sjukhus	10	148.2
Karlsborg	KSS Allergimott (ÖNH, Barn)	14	148.0
Heby	Akademiska sjukhuset	28	145.1
Robertsfors	Norrlands US	13	142.9
Tranås	Höglandssjukhuset	37	141.6
Årjäng	Arvika Sjukhus	19	141.6
Boxholm	US i Linköping/Allergi Centrum	11	141.5
Ockelbo	Länssjuk. Gävle/Sandviken	11	140.9
Hultsfred	Oskarshamns lasarett	27	140.3
Mellerud	Lidköpings Sjukhus	18	139.9
Motala	US i Linköping/Allergi Centrum	85	138.1
Hylte	Halmstads länssjukhus	21	137.8
Mariestad	KSS Allergimott (ÖNH, Barn)	48	137.7
Hofors	Avesta lasarett	19	136.6
Kramfors	Härnösand sjukhus	35	135.4
Tingsryd	Blekingesjukhuser, Karlshamn	24	134.4
Borgholm	Oskarshamns lasarett	21	133.5
Markaryd	Ljungby lasarett	20	130.8
Tranemo	SÄS, Borås Lasarett	23	130.5
Sävsjö	Sjukhuset Värnamo	23	129.7
Säffle	Centrallasarett Karlstad	30	129.6
Köping	Lindesberg Sjukhus	51	129.2
Sollefteå	Sollefteå sjukhus	36	128.9
Åtvidaberg	US i Linköping/Allergi Centrum	22	128.2
Högsby	Oskarshamns lasarett	11	125.9
Vetlanda	Höglandssjukhuset	54	125.5

Municipality	Nearest specialized AR clinic	Population receiving AIT for AR*	Round-trip distance to clinic (km)**
Leksand	Mora	31	124.8
Nybro	Länssjukhuset i Kalmar	39	124.2
Falköping	KSS Allergimott (ÖNH, Barn)	65	124.1
Sotenäs	Uddevalla sjukhus	18	123.3
Skinnskatteberg	Lindesberg Sjukhus	9	118.8
Hällefors	Lindesberg Sjukhus	13	118.2
Nordanstig	Hudiksvalls sjukhus	18	117.7
Gislaved	Sjukhuset Värnamo	57	117.5
Arboga	Regionsjuk. Örebro ÖNH	27	116.9
Orsa	Mora	14	116.6
Örnsköldsvik	Örnsköldsvik	108	116.3
Tjörn	Angered Närsjukhus	32	115.7
Finspång	Kullbergskasjukhuset/Katrineholm	42	115.4
Gagnef	Borlänge sjukhus	20	113.1
Herrljunga	SÄS, Borås Lasarett	18	112.9
Hallsberg	Regionsjuk. Örebro ÖNH	32	112.6
Vaggeryd	Länssjuk. Ryhov, Jönköping	29	112.5
Essunga	NÄL Trollhättan	11	112.0
Tidaholm	KSS Allergimott (ÖNH, Barn)	25	111.8
Vännäs	Norrlands US	18	109.4
Gnesta	Södertälje BUMM	22	108.0
Flen	Kullbergskasjukhuset/Katrineholm	32	107.7
Torsås	Blekingesjukhuset, Karlskrona	14	107.1
Töreboda	KSS Allergimott (ÖNH, Barn)	18	106.6
Strängnäs	Eskilstuna	74	106.4
Munkedal	Uddevalla sjukhus	21	106.1
Vårgårda	SÄS, Borås Lasarett	24	105.5
Enköping	Västerås	92	104.8
Surahammar	Västerås	20	104.6
Örkelljunga	Hässleholms lasarett	20	103.9
Ulricehamn	SÄS, Borås Lasarett	49	103.4
Laholm	Halmstads länssjukhus	51	101.7
Filipstad	Hagfors VC	20	101.5
Älmhult	Ljungby lasarett	35	101.4
Stenungsund	Angered Närsjukhus	54	100.6
Gotland	Visby lasarett	118	100.2
Hjo	KSS Allergimott (ÖNH, Barn)	18	99.7
Sandviken	Länssjuk. Gävle/Sandviken	76	99.5
Lessebo	Centrallasarettet i Växjö	17	99.5
Osby	Hässleholms lasarett	26	99.1
Mullsjö	Länssjuk. Ryhov, Jönköping	14	99.0
Sunne	Torsby Sjukhus	26	98.8
Vara	Lidköpings Sjukhus	31	97.8

Municipality	Nearest specialized AR clinic	Population receiving AIT for AR*	Round-trip distance to clinic (km)**
Forshaga	Karlstad Privat P.O.W.	23	97.7
Trosa	Nyköpings lasarett	29	97.6
Ludvika	Ludvika lasarett	51	97.2
Eda	Arvika Sjukhus	17	96.7
Fagersta	Avesta lasarett	26	96.6
Färgelanda	Uddevalla sjukhus	13	96.6
Hörby	Specialisthuset Eslöv	31	96.5
Munkfors	Hagfors VC	7	96.0
Klippan	Sjukhuset i Ängelholm	35	95.4
Håbo	Akademiska sjukhuset	44	94.9
Ljusnarsberg	Ludvika lasarett	9	94.8
Ydre	Höglandssjukhuset	7	94.7
Mjölby	US i Linköping/Allergi Centrum	55	93.6
Svenljunga	Skene BUMM	21	92.1
Hedemora	Avesta lasarett	30	91.3
Kil	Centrallasarett Karlstad	24	90.9
Östra Göinge	Hässleholms lasarett	29	90.6
Perstorp	Hässleholms lasarett	15	87.9
Sundsvall	Sundsvalls sjukhus	193	87.8
Kungsör	Eskilstuna	17	87.8
Ronneby	Blekingesjukhuser, Karlshamn	57	87.5
Sigtuna	Akademiska sjukhuset	98	87.5
Alingsås	Lerum BUM	81	87.4
Grums	Centrallasarett Karlstad	18	86.0
Lysekil	Uddevalla sjukhus	28	85.6
Orust	Uddevalla sjukhus	30	84.4
Gnosjö	Sjukhuset Värnamo	19	83.8
Timrå	Sundsvalls sjukhus	35	83.6
Nässjö	Höglandssjukhuset	62	81.8
Lekeberg	Karlskoga Lasarett	17	81.4
Habo	Länssjuk. Ryhov, Jönköping	25	81.3
Sala	Avesta lasarett	45	80.5
Vallentuna	Danderyds sjukhus	67	79.4
Olofström	Blekingesjukhuser, Karlshamn	26	79.3
Mörbylånga	Länssjukhuset i Kalmar	31	78.6
Aneby	Höglandssjukhuset	13	78.4
Mönsterås	Oskarshamns lasarett	26	77.9
Skara	Lidköpings Sjukhus	36	77.8
Alvesta	Centrallasarettet i Växjö	39	77.5
Söderköping	Norrköpings lasarett	29	77.2
Sjöbo	Ystad lasarett	38	76.7
Tibro	KSS Allergimott (ÖNH, Barn)	22	75.6
Grästorp	NÄL Trollhättan	11	74.4

Municipality	Nearest specialized AR clinic	Population receiving AIT for AR*	Round-trip distance to clinic (km)**
Älvkarleby	Länssjuk. Gävle/Sandviken	19	74.2
Lilla Edet	NÄL Trollhättan	28	73.3
Tomelilla	Simrishamns sjukhus	27	72.9
Bromölla	Blekingesjukhuser, Karlshamn	24	72.5
Falkenberg	Spec. vården Falkenberg	91	71.8
Upplands-Bro	Lidingö BUMM	61	70.0
Ale	Angered Närsjukhus	63	69.9
Österåker	Fysikalisk medicin	94	69.6
Värmdö	Nacka sjukhus	90	69.4
Hallstahammar	Västerås	32	69.4
Vingåker	Kullbergskasjukhuset/Katrineholm	18	69.2
Storfors	Kristinehamns sjukhus	8	67.7
Västervik	Västerviks lasarett	71	67.5
Östersund	Östersunds sjukhus	125	67.0
Malmö	US i Lund	686	67.0
Degerfors	Karlskoga Lasarett	18	66.9
Kumla	Regionsjuk. Örebro ÖNH	43	66.7
Nora	Lindesberg Sjukhus	21	66.4
Falun	Falu lasarett	116	66.1
Bollebygd	Lerum BUM	19	65.3
Hammarö	Centrallasarett Karlstad	33	65.0
Götene	Lidköpings Sjukhus	26	64.6
Säter	Borlänge sjukhus	22	63.5
Karlstad	Karlstad Privat P.O.W.	186	61.5
Svedala	Sjukhuset Trelleborg	45	61.5
Öckerö	Backa BUM	25	61.1
Skellefteå	Skellefteå	143	60.6
Höör	Specialisthuset Eslöv	33	59.6
Båstad	Sjukhuset i Ängelholm	31	58.8
Smedjebacken	Ludvika lasarett	21	58.7
Bjuv	Helsinborgs lasarett	31	58.5
Lund	Spartakliniken	248	58.3
Ekerö	Södertälje BUMM	57	57.5
Norberg	Avesta lasarett	11	57.5
Skurup	Ystad lasarett	32	55.7
Knivsta	Akademiska sjukhuset	39	55.6
Mora	Mora	40	54.6
Sölvesborg	Blekingesjukhuser, Karlshamn	34	54.4
Eksjö	Höglandssjukhuset	35	54.4
Hudiksvall	Hudiksvalls sjukhus	73	53.4
Umeå	Norrlands US	255	53.1
Svalöv	Specialisthuset Eslöv	28	52.8
Åstorp	Sjukhuset i Ängelholm	32	51.9

Municipality	Nearest specialized AR clinic	Population receiving AIT for AR*	Round-trip distance to clinic (km)**
Vänersborg	NÄL Trollhättan	77	50.2
Lycksele	Lycksele	24	48.9
Kungälv	Angered Närsjukhus	94	47.9
Uppsala	Enköpings barnspec mott	463	46.0
Härnösand	Härnösand sjukhus	48	45.8
Luleå	Sunderbyn	153	45.6
Karlskrona	Blekingesjukhuset, Karlskrona	129	45.5
Nykvarn	Södertälje BUMM	22	45.3
Trollhättan	NÄL Trollhättan	115	44.7
Avesta	Avesta lasarett	44	44.0
Kävlinge	US i Lund	63	43.5
Höganäs	Sjukhuset i Ängelholm	54	41.6
Oskarshamn	Oskarshamns lasarett	53	40.8
Jönköping	Länssjuk. Ryhov, Jönköping	279	40.8
Vellinge	Sjukhuset Trelleborg	73	40.6
Burlöv	US i Lund	38	40.2
Varberg	Varbergs lasarett	130	39.9
Upplands-Väsby	Lidingö BUMM	94	39.9
Oxelösund	Nyköpings lasarett	24	38.6
Härryda	Mölnlycke BUMM	76	38.4
Lidköping	Lidköpings Sjukhus	79	38.3
Norrtälje	Norrtälje sjukhus	126	38.2
Halmstad	Halmstads länssjukhus	203	35.2
Arvika	Arvika Sjukhus	50	34.1
Eskilstuna	Eskilstuna	209	34.0
Kungsbacka	Kungsbacka sjukhus	166	33.8
Tyresö	Sachska/Handen BUMM	95	33.7
Lerum	Lerum BUM	84	30.5
Norrköping	Norrköpings lasarett	281	30.3
Ljungby	Ljungby lasarett	55	29.6
Vaxholm	Fysikalisk medicin	23	29.5
Botkyrka	Huddinge	185	28.9
Karlshamn	Blekingesjukhuser, Karlshamn	63	28.2
Ängelholm	Sjukhuset i Ängelholm	85	28.0
Kristinehamn	Kristinehamns sjukhus	47	27.4
Karlskoga	Karlskoga Lasarett	59	27.3
Täby	Danderyds sjukhus	144	27.1
Staffanstorp	Spartakliniken	51	26.8
Örebro	Regionsjuk. Örebro ÖNH	305	26.5
Trelleborg	Sjukhuset Trelleborg	90	26.3
Kalmar	Länssjukhuset i Kalmar	139	26.2
Järfälla	Lidingö BUMM	162	25.9
Haninge	Sachska/Handen BUMM	187	25.6

**Municipality**	**Nearest specialized AR clinic**	**Population receiving AIT for AR***	**Round-trip distance to clinic (km)****
Lomma	US i Lund	48	25.6
Lindesberg	Lindesberg Sjukhus	46	25.3
Växjö	Centrallasarettet i Växjö	187	24.9
Uddevalla	Uddevalla sjukhus	111	24.8
Nynäshamn	Idun Barn/Ungdomsmott.	58	24.1
Södertälje	Södertälje BUMM	197	23.4
Katrineholm	Kullbergskasjukhuset/Katrineholm	67	23.3
Hagfors	Hagfors VC	22	22.4
Simrishamn	Simrishamns sjukhus	37	22.0
Nyköping	Nyköpings lasarett	112	22.0
Salem	Södertälje BUMM	34	21.5
Linköping	US i Linköping/Allergi Centrum	322	20.9
Värnamo	Sjukhuset Värnamo	67	20.4
Mölndal	Mölnlycke BUMM	136	20.1
Mark	Skene BUMM	68	19.5
Borlänge	Borlänge sjukhus	102	19.3
Helsingborg	Helsinborgs lasarett	292	18.5
Skövde	KSS Allergimott (ÖNH, Barn)	111	15.5
Ystad	Ystad lasarett	61	14.9
Sundbyberg	Lidingö BUMM	104	14.5
Huddinge	Sachska/Farsta BUMM	222	14.5
Landskrona	Landskrona lasarett	90	13.2
Sollentuna	Lidingö BUMM	146	13.2
Partille	Sävedalen BUM	77	12.5
Hässleholm	Hässleholms lasarett	102	12.4
Gävle	Länssjuk. Gävle/Sandviken	201	10.2
Västerås	Västerås	305	9.1
Borås	SÄS, Borås Lasarett	222	6.8
Solna	Karolinska Universitetsjukhuset Solna	164	6.4
Stockholm	St. Görans sjukhus	1904	6.3
Danderyd	Danderyds sjukhus	64	6.2
Lidingö	Fysikalisk medicin	94	5.7
Eslöv	Specialisthuset Eslöv	67	5.3
Göteborg	Läkarhuset +7	1145	4.6
Kristianstad	Sjukhuset i Kristianstad	168	4.2
Nacka	Nacka sjukhus	210	3.8

Abbreviations: AIT, Allergen immunotherapy; AR, Allergic rhinitis.

*Patient figures rounded to the nearest whole number

**Results arranged based on travel distances, from largest travel distances to shortest travel distances
